# Speech Perception Development From Childhood to Adulthood Following Pediatric Cochlear Implantation: A 30-year Longitudinal Study

**DOI:** 10.1016/j.jpedcp.2026.200213

**Published:** 2026-04-30

**Authors:** Chieh Kao, Kathryn B. Wiseman, Sophie E. Ambrose, Jacquelyn L. Baudhuin, Adam K. Bosen, Amberlee Haggerty, Melissa R. Henry, Kristen L. Janky, Elizabeth A. Kelly, Jessie N. Patterson, Jeffrey L. Simmons, Victoria Sweeney, Z. Ellen Peng

**Affiliations:** 1 Boys Town National Research Hospital, Omaha, NE; 2 National Taipei University of Nursing and Health Sciences, Taipei, Taiwan

**Keywords:** age at implantation, etiologies of hearing loss, longitudinal outcomes, pediatric cochlear implant users, socioeconomic status, speech perception development

## Abstract

**Objective:**

Speech perception development in pediatric cochlear implant users varies widely, and its progression into late childhood and early adulthood remains unclear. This study examines long-term developmental trajectories and influencing factors using a longitudinal clinical dataset.

**Study design:**

Speech perception data collected across 22 clinical tests conducted in English and in quiet were retrospectively analyzed with a hierarchical scoring system to understand development of 294 pediatric cochlear implant users between 1992 and 2024 at Boys Town National Research Hospital. Medical records provided data on age at cochlear implant activation, year of first cochlear implant implantation, device configuration, daily device use, hearing loss etiology, revision history, primary language use, insurance type, race, and comorbidities. These variables were incorporated into statistical models to predict longitudinal speech perception outcomes.

**Results:**

Pediatric cochlear implant users generally show steady improvement in speech perception with chronological age. Faster early development of speech perception was associated with implantation before 18 months of age, bilateral cochlear implant use, longer daily device use, spoken English as a primary language, having private insurance, and better vestibular function. Alternatively, auditory neuropathy, syndromic hearing loss, hearing loss due to prenatal infections and low socioeconomic status were associated with poorer speech perception.

**Conclusions:**

Auditory neuropathy or syndromic hearing loss and low socioeconomic status are key factors associated with slower speech perception development. Improving access to cochlear implant specialists and encouraging consistent device use—especially for later implantees—may enhance outcomes. Additional support for families and educators is recommended to promote increased device use.

Cochlear implants are a transformative intervention for children with severe to profound sensorineural hearing loss. However, speech perception development varies widely due to neurobiological, medical, rehabilitative, and social constraints.^1^, ^2^, ^3^ Individual factors such as timing of auditory stimulation,^4^ medical conditions,^5^ device use,^6^ and access to aural rehabilitation^7^ jointly shape developmental trajectories in pediatric cochlear implant users. To monitor postimplantation communication outcomes and guide cochlear implant programming, speech perception testing is routinely used in cochlear implant clinics.

Speech perception outcomes are highly variable among children with cochlear implants, influenced by audiologic (eg, age at implantation),^2^^,^^4^^,^^8^^,^^9^ medical (eg, etiology of hearing loss,^5^^,^^10^^,^^11^ and comorbidity),^12^, ^13^, ^14^ device-related (eg, device use^15^, ^16^, ^17^ and device configuration),^18^, ^19^, ^20^, ^21^ and demographic (eg, socioeconomic status [SES]^1^^,^^12^^,^^22^^,^^23^ and primary language) factors.^23^ Early implantation consistently supports faster development of speech perception^4^^,^^12^^,^^24^^,^^25^ and spoken language.^22^ Vestibular and balance dysfunction, common in children with severe to profound hearing loss, may further complicate aural habilitation after implantation of cochlear implants.^26^^,^^27^ Previous longitudinal studies primarily focused on cochlear implant users’ speech perception development during limited postactivation periods in childhood,^1^, ^2^, ^3^^,^^24^^,^^25^^,^^28^^,^^29^ with few longitudinal studies comparing outcomes across age-adjusted clinical speech tests.^29^

Some cross-sectional data on pediatric cochlear implant users’ speech perception outcomes examined the impact of hearing loss etiology. Those with genetic mutations that only involve hearing loss tend to perform better on speech tasks.^10^ Children with a small or missing hearing nerve (auditory nerve deficiency or hypoplasia) often show poorer speech perception outcomes and need visual cues to aid communication.^30^, ^31^, ^32^ Prenatal (eg, cytomegalovirus)^5^ and postnatal infections (eg, meningitis)^10^ are also linked to slower speech perception progression after receiving cochlear implants. Therefore, the current study of longitudinal speech perception development should include hearing loss etiology to address its long-term impact on speech perception outcomes in pediatric cochlear implant users.

In addition to the cause of hearing loss, the type of device a child uses also changes how well they hear and may have long-term effects on their speech perception. Children with bilateral cochlear implants or bimodal devices (ie, cochlear implant and hearing aid) generally perform better in noisy environments,^28^ where most real-world listening occurs, than those with a unilateral cochlear implant (ie, bilateral hearing loss with 1 cochlear implant and no device on the other ear). Because the practice of bilateral implantation emerged later, the bilateral advantage can potentially be explained by better device technologies. For this reason, the current study also includes device type and the year pediatric patients received their cochlear implant to examine longitudinal effects from device-related factors on speech perception.

Beyond medical and device-related considerations, demographic factors have received increasing attention for their role in shaping the development of speech perception. Higher SES and parental education are associated with better speech outcomes,^1^^,^^22^ whereas disadvantaged backgrounds may slow progress,^12^^,^^23^ partly through differences in parental responsiveness toward their children and support for communication.^1^^,^^33^ Children whose primary home language differs from the mainstream language often show slower developmental rate for speech perception,^23^ reflecting interactions with rehabilitation, educational context, and daily language exposure. Finally, consistent daily cochlear implant use, which usually relies on parental/familial support, is strongly associated with better speech perception in quiet^34^^,^^35^ and often predicts speech performance more robustly than demographic factors.^36^^,^^37^

The current study examined a 30-year longitudinal clinical speech dataset collected across 22 speech tests in quiet with varying difficulty levels after pediatric implantation. This dataset has been maintained by the Cochlear Implant Clinic at Boys Town National Research Hospital (BTNRH). We aimed to identify individual factors associated with delayed speech perception in quiet and potential factors associated with better outcomes to guide clinical counseling on modifiable variables.

## Methods

### Participants

This retrospective longitudinal cohort study included 294 cochlear implant users whose cochlear implants were activated before age 18, excluding those with no speech perception testing records. Patients included in this study were followed clinically by the cochlear implant audiologists at the BTNRH cochlear implant clinic. The clinic serves both pediatric and adult patients and includes an interdisciplinary team of otolaryngologists who perform implantation in-house, cochlear implant audiologists, vestibular audiologists, speech-language pathologists, and early intervention specialists. Patients in this study received their implants between 1992 and 2024. The mean age at first cochlear implant activation was 4.0 ± 4.1 years (range, 0.7-17.6 years). Audiologic, device, demographic, and medical data are summarized in Table I. The study was approved by the Institutional Review Board at BTNRH (#21-19-XP).

**Table I:** The 22 clinical speech tests, their base scores assigned based on their difficulty levels, and the count of each test

Name of the test	Type of the test	Base score	Count
IT-MAIS/MAIS	Questionnaires	0	104
LittlEARS	Questionnaires	0	56
ESP low verbal pattern perception	Closed-set patterns or words	100	18
ESP low verbal spondee	Closed-set patterns or words	200	10
ESP low verbal Monosyllable	Closed-set patterns or words	300	15
TAC-4	Closed-set patterns or words	400	33
NU-CHIPS	Closed-set patterns or words	400	134
Mr. Potato Head	Closed-set phrases or sentences	400	19
WIPI	Closed-set patterns or words	500	149
TAC-5	Closed-set phrases or sentences	500	33
Common Phrases	Open-set phrases or sentences	500	58
TAC-6	Closed-set phrases or sentences	600	35
MLNT-Easy/Hard	Open-set words	600	17
LNT-Easy/Hard	Open-set words	700	17
NU-CHIPS	Open-set words	700	2
WIPI	Open-set words	700	5
PBK	Open-set words	800	267
HINT	Open-set sentences	800	229
HINT-C	Open-set sentences	800	180
NU-6	Open-set words	900	1
CNC	Open-set words	900	305
BabyBio	Open-set sentences	900	341
AzBio	Open-set sentences	1000	639
PRESTO	Open-set sentences	1100	81

*AzBio*, a sentence recognition test developed in the Department of Speech and Hearing Science at Arizona State University; *BabyBio*, Pediatric AzBio Sentence Test; *CNC*, Consonant-Nucleus-Consonant; *HINT*, Hearing in Noise Test; *HINT-C*, Hearing in Noise Test for Children; *IT-MAIS*, Infant-Toddler Meaningful Auditory Integration Scale; *ESP*, Early Speech Perception; *LittlEARS*, Little Ears Auditory Questionnaires; *LNT*, Lexical Neighborhood Test; *MAIS*, Meaningful Auditory Integration Scale; *MLNT*, Multisyllabic Lexical Neighborhood Test; *NU-6*, Northwestern University Auditory Test Number 6; *NU-CHIPS*, Northwestern University-Children's Perception of Speech; *PBK*, Phonetically Balanced Kindergarten; *PRESTO*, Perceptually Robust English Sentence Test Open-set; *TAC*, Test of Auditory Comprehension; *WIPI*, Word Intelligibility by Picture Identification.

Word- or keyword-level scoring was used for each test.

### Speech Perception Scores

At each cochlear implant programming visit, speech perception testing in quiet in North American English (without visual cues) was conducted and recorded in a clinical database. The number of appointments per participant ranged from 1 to 26 (mean, 9 ± 6), with follow-up duration from 0 to 24 years (mean, 8.7 ± 6.3) (Supplementary Figure S1, available at www.jpeds.com). Tests were selected by audiologists based on patients’ speech and language skills at each appointment. Age at testing ranged from 0.9 to 41.9 years, and time since cochlear implant activation ranged from 0.1 to 34.6 years. To enable comparisons across different skill-adjusted tests, we applied a hierarchical speech scoring system adapted from prior studies^29^^,^^38^ and clinical consensus (Table I). Each test was assigned a base score reflecting skill difficulty (eg, base score for questionnaire = 0, base score for test of close-set pattern recognition = 100, etc), and a hierarchical score was calculated as word-level percent correct plus the base score (eg, 68% on a test with a base score of 400 yields a score of 468). When multiple tests were completed at 1 visit, the highest hierarchical score was used. The final dataset comprised 2748 speech scores in quiet from 294 participants with cochlear implants.

### Individual Factors

Audiologic (age at first activation of the cochlear implant, cochlear implant experience), device (device configuration, revision history, implantation year to account for era effect), demographics (primary language, race/ethnicity, insurance type as a proxy for SES), and medical (etiology, speech or language delay, vestibular involvement, number of complex diagnoses) variables were extracted from medical records (Table II). Factors that are time sensitive or time varying (eg, device configuration, insurance type, device use, onset of comorbidities) were modeled as fixed effects using the most recently available medical records. Daily device use was obtained from cochlear implant/hearing aid datalogging, with the higher value retained for bilateral or bimodal users. A subset of participants completed receptive vocabulary (Peabody Picture Vocabulary Test)^39^ and cognitive (Wechsler Abbreviated Scale of Intelligence)^40^ assessments during separate research visits. These data were appended from a research database.

**Table II:** Count of patient's audiologic, demographic, device, and medical information

Factors	Count
Audiologic factor	
First cochlear implant age of activation	
Early (<18 months)	109
Middle (between 18 and 36 months)	75
Late (>36 months)	110
Demographic factors	
Biological sex	
Female	142
Male	152
Race/ethnicity	
Asian	5
Black	9
Latino/Hispanic	3
White	269
Unknown	8
Primary language	
ASL	30
English	243
Spanish	21
Insurance type	
Government	155
Private	139
Daily device use, hours	
<6	32
6-9	35
9-12	22
>12	122
No available datalogging records	83
Device factors	
Hearing configuration	
Bilateral cochlear implants (simultaneous and sequential)	155
Bimodal (cochlear implant and hearing aid)	61
Single-sided deafness with a cochlear implant	12
Unilateral cochlear implant	66
Revision history	
Yes	67
No	227

Medical factors	Count	Age at first cochlear implant activation		
	All	Early	Middle	Late
Etiology of hearing loss				
Auditory neuropathy	16	3	5	8
Cochleovestibular malformation	21	3	5	13
Cochlear nerve deficiency	2	1	1	0
Genetic nonsyndromic hearing loss	26	23	2	1
Genetic syndromic hearing loss	24	13	5	6
Postnatal infection	15	4	5	6
Prenatal infection	19	9	5	5
Multiple etiologies	2	2	0	0
Unknown	169	51	47	71
Diagnosis of speech delay				
Never	234			
At least once	60			
No. of other complex diagnoses				
None	193			
1	64			
2	25			
>3	12			
Diagnosis of vestibular involvement				
Did not test	124			
Normal	110			
Unilateral vestibular involvement	39			
Bilateral vestibular involvement	21			

*ASL*, American Sign Language; *HA*, hearing aid; *SSD*, single-sided deafness.

### Statistical Analysis—Primary

Linear mixed-effect (LME) models were used to examine factors associated with longitudinal speech-in-quiet perception development, accounting for within-participant correlation. An initial model including all variables of interest was fit, followed by backward stepwise selection for model optimization. Fixed effects included chronological age, cochlear implant experience, year of first cochlear implant implantation, first cochlear implant age of activation (<18 months, 18-36 months, >36 months), device configuration, daily device use (<6, 6-9, 9-12, >12 hours), etiology, revision history, primary language, insurance type, race/ethnicity, speech or language delay, vestibular involvement, number of complex diagnoses, Peabody Picture Vocabulary Test scores, and Wechsler Abbreviated Scale of Intelligence scores. Participant was entered as the random intercept. Interactions of interest included 3-way interactions between chronological age, age at first activation, and primary language or etiology, along with corresponding 2-way interactions.

### Statistical Analysis—Secondary

Given the established importance of daily cochlear implant use, secondary analyses examined daily device use as a mediator of the relationships between demographic variables and speech perception outcomes. All analyses were conducted in R (version 4.4.1) using RStudio (version 2023.09.1, Build 494), with packages including “buildmer”^41^ (v2.11) and “lme4”^42^ (v1.1.36). Supplementary Figure S2, available at www.jpeds.com provides an analysis of data without patients who were lost to follow-up within 1 year.

## Results

### Primary Analysis Model

**Figure 1:**
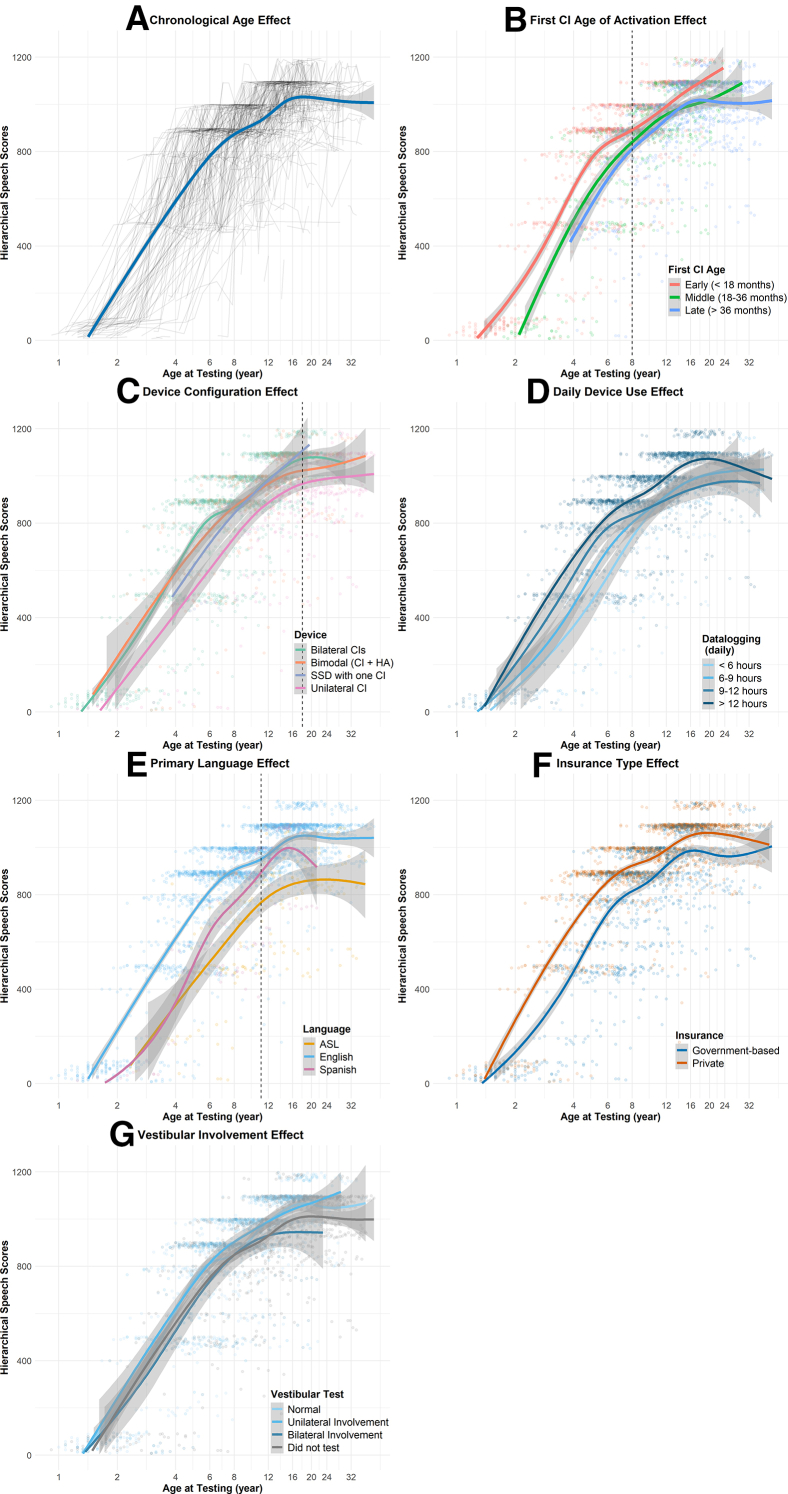
Hierarchical score of speech in quiet perception as a function of log-transformed age in years (**A-F**), illustrating effects of (**B**) first cochlear implant age of activation, (**C**) device configuration, (**D**) daily device use, (**E**) primary language, (**F**) insurance type, and (**G**) vestibular status on the development of speech perception. Curve fits with shaded area indicate confidence intervals adjusted to allow pairwise comparisons. Dashed lines were added to highlight the age at which overlapping speech perception performance was observed among groups.

**Figure 2:**
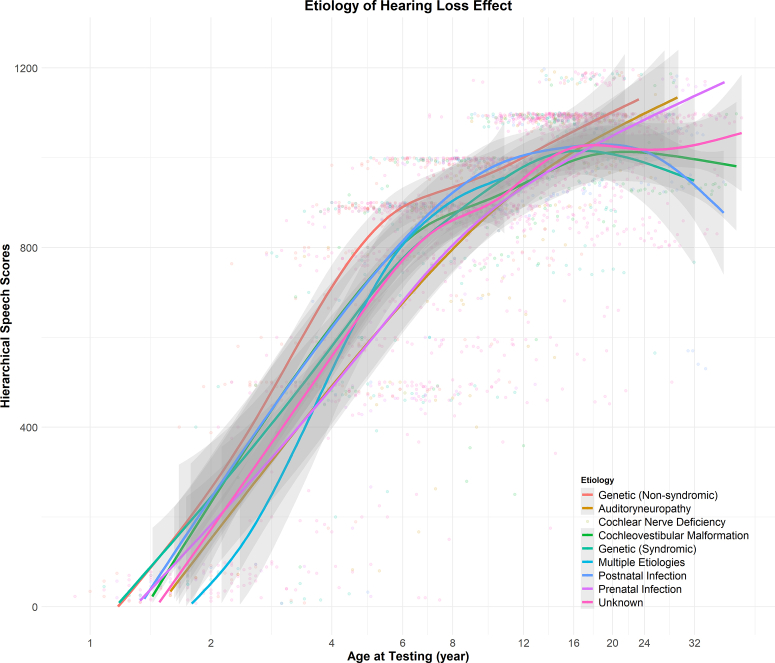
The interaction between chronological age (log-transformed) and etiology of hearing loss on speech perception scores.

**Figure 3:**
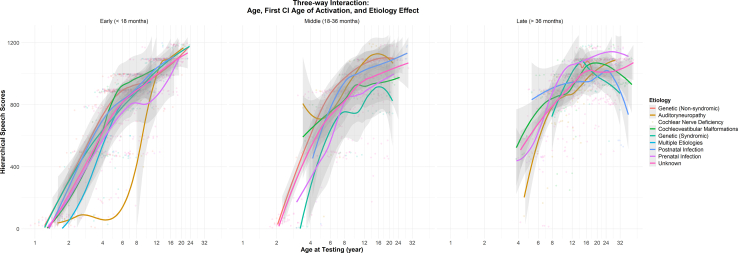
The 3-way interaction between chronological age, first cochlear implant age of activation, and etiology of hearing loss on speech perception scores.

**Figure 4:**
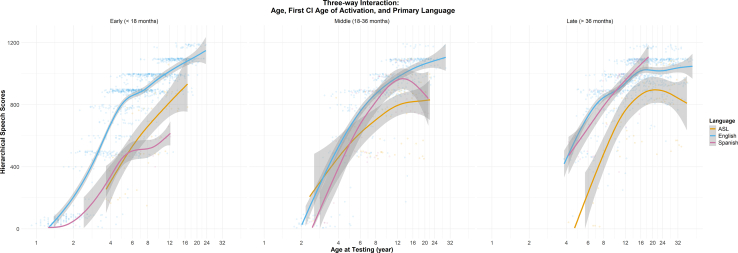
The 3-way interaction between chronological age, first cochlear implant age of activation, and etiology of hearing loss on speech perception scores.

Longitudinal changes in hierarchical speech-in-quiet scores were analyzed using an LME model, with individual factors of interest (see the Appendix and the Supplementary Table, available at www.jpeds.com) entered as fixed effects and participant as a random intercept. Model optimization was performed using the “buildmer”^41^ package to reduce model dimensions. The final model revealed significant main effects of chronological age, *F*(1, 1295) = 1796, ${ƞ}^{2}$ = 0.58, *P* < .001 (Figure 1A), first cochlear implant age of activation, *F*(2, 595) = 5.14, ${ƞ}^{2}$ = 0.02, *P* = .006 (Figure 1B), device configuration, *F*(3, 157) = 15.66, ${ƞ}^{2}$ = 0.23, *P* < .001 (Figure 1C), daily device use, *F*(3, 154) = 19.03, ${ƞ}^{2}$ = 0.27, *P* < .001 (Figure 1D), primary language, *F*(2, 598) = 34.94, ${ƞ}^{2}$ = 0.11, *P* < .001 (Figure 1E), insurance type, *F*(1, 145) = 30.54, ${ƞ}^{2}$ = 0.17, *P* < .001 (Figure 1F), and vestibular involvement, *F*(3, 145) = 4.5, ${ƞ}^{2}$ = 0.09, *P* = .004 (Figure 1G). Significant 2-way interactions were observed between chronological age and first cochlear implant age of activation, *F*(2, 1627) = 287.88, *P* < .001 (Figure 1B), chronological age and etiology, *F*(8, 1238) = 5.39, *P* < .001 (Figure 2), chronological age and primary language, *F*(2, 1624) = 45.16, *P* < .001 (Figure 1E), and age at first cochlear implant activation and etiology, *F*(12, 397) = 2.44, *P* = .004 (Figure 3). Significant 3-way interactions include chronological age and age at first cochlear implant activation with etiology of hearing loss, *F*(12, 1671) = 3.45, *P* < .001 (Figure 3), and between chronological age and age at first cochlear implant activation with primary language, *F*(4, 1783) = 3.43, *P* = .008 (Figure 4). The final model explained 61% of the variance in speech scores attributable to fixed effects (marginal *R*^*2*^ = 0.61), increasing to 76% with inclusion of random effects (conditional *R*^*2*^ = 0.76).

### Secondary Mediation Analysis

Among the 222 participants with available datalogging records, secondary analyses examined daily cochlear implant use as a mediator of speech-in-quiet perception outcomes. The dataset was restricted to each participant's most recent datalogging record and the corresponding hierarchical speech score. Following steps outlined by Baron and Kenny,^43^ we screened for significant prediction of individual variables on the speech score (step 1 models in Table II) and daily cochlear implant use (step 2 models in Table III), separately. Age at testing and age at first cochlear implant activation (in years) were identified as significant predictors of both speech scores and daily cochlear implant use. In mediation models including both age variables and daily cochlear implant use (step 3 model in Table III), daily cochlear implant use emerged as a partial mediator of the relationship between chronological age and speech-in-quiet score, and a full mediator of the relationship between age at first cochlear implant activation and speech-in-quiet score.

**Table III:** Stepwise model testing daily cochlear implant use as a mediator

IV	Estimate	*t*	*P* value
Step 1: IV > most recent speech score			
Age at time of speech testing	12.49	7.39	<.001
Age at first cochlear implant activation	0.61	1.98	.049
Step 2: IV > daily cochlear implant use (mediator)			
Age at time of speech testing	0.083	2.23	.027
Age at first cochlear implant activation	−0.015	−2.4	.017
Step 3: both IVs + mediator > most recent speech score			
Age at time of speech testing	11.30	6.00	<.001
Age at first cochlear implant activation	−0.071	−0.23	.82
Daily cochlear implant use	16.92	5.68	<.001

*IV*, Independent variable.

A follow-up mediation analysis using nonparametric bootstrapping (1000 simulations) with R package “mediation”^44^ (v4.5.0) quantified the mediation effect sizes. The combined direct effect of the 2 age variables on speech-in-quiet score was significant (*β* = 11.30; 95% CI, 7.30-15.44; *P* < .0001), as was the indirect effect through daily cochlear implant use (*β* = 2.85; 95% CI, 1.19-4.99; *P* < .0001). Together, these results indicate that daily cochlear implant use fully mediates the effect of age at first cochlear implant activation and partially mediates the effect of chronological age on pediatric cochlear implant users' outcomes of speech perception in quiet.

## Discussion

In this study, we analyzed a large longitudinal clinical dataset spanning >30 years of routine speech-in-quiet testing in pediatric cochlear implant users at BTNRH to identify factors associated with speech-in-quiet perception development. Several factors significantly predicted developmental trajectory beyond chronological age.

Daily device use emerged as the strongest predictor of speech in-quiet perception development (${ƞ}^{2}$ = 0.27) (Figure 1D), with longer daily use associated with faster progression to more difficult speech materials. Children using their devices >12 hours per day reached open-set word recognition (eg, PBK) approximately 2.5 years earlier than those with <6 hours of daily device use. This finding aligns with prior studies demonstrating the importance of consistent auditory input and supports the view that device use is a key, modifiable contributor to outcomes.^6^^,^^17^^,^^34^^,^^35^ Notably, daily device use fully mediates the benefits of early implantation, suggesting that early implantation alone may be insufficient without consistent device use. Although causality cannot be inferred, the relationship between device use and outcomes is likely bidirectional, underscoring the clinical importance of counseling to promote consistent device use.

Device configuration is the second strongest predictor of speech-in-quiet perception development (${ƞ}^{2}$ = 0.23) (Figure 1C), with children using bilateral cochlear implants demonstrating better and faster speech-in-quiet perception development than unilateral cochlear implant users by reaching open-set word recognition almost 4 years earlier. Because the year of first cochlear implant implantation (differences in which would reflect differences in cochlear implant technology) did not significantly explain the data variance, this advantage may reflect the benefits of bilateral electrical stimulation over unilateral cochlear implants (the contralateral ear with severe-to-profound hearing loss without a hearing device), supporting clinical efforts toward bilateral implantation.^45^ Children with bimodal devices or single-sided deafness showed developmental trajectories comparable with those with bilateral cochlear implants, indicating that residual acoustic hearing in the contralateral ear does not impede speech-in-quiet perception development.

Insurance type, used as a proxy for SES, is the third strongest predictor of speech-in-quiet perception development (${ƞ}^{2}$ = 0.17) (Figure 1F).^36^ Children with private insurance show earlier emergence of open-set word recognition ability by 2.5 years than those with government-funded insurance, and this performance gap persists into early adulthood. This finding suggests that persistent disparities are linked to family resources after device implantation.^7^^,^^46^ Beyond this dataset, there are children of lower SES who meet the criteria for implantation but did not receive an implant.^47^ Further research is necessary to understand whether modifiable aspects of SES, such as improved access to pediatric hearing healthcare,^48^ specialized services for patients on government-funded insurance,^49^^,^^50^ and family-centered care, could help to mitigate these long-term disparities.

Several meaningful predictors with smaller effect sizes are identified. Spanish-speaking children show lower speech scores than English-speaking peers until approximately 12 years of age. However, this finding should be interpreted cautiously, given that all clinical speech tests were administered in English.^51^^,^^52^ Similarly, lower speech-in-quiet perception scores among sign language users do not necessarily reflect their broader language competency, but rather their speech perception performance (plateaus at simpler open-set sentences) without visual or gesture support (Figure 1E). Children with bilateral vestibular involvement demonstrated poorer speech perception in quiet than those with unilateral or no vestibular involvement, a novel finding that may relate to balance dysfunction,^26^^,^^27^^,^^53^ comorbidities,^54^ altered early social interactions,^55^ increased rehabilitation needs, or higher cognitive load^56^ during development.

Children with certain etiologies can experience differential rates of speech-in-quiet development during sensitive periods (Figure 2). Children with auditory neuropathy show delayed onset of speech-in-quiet perception development until 8 years of age (Figure 3) despite early implantation. Children with syndromic hearing loss or prenatal infections (eg, congenital cytomegalovirus infection) exhibit poorer speech outcomes than children with nonsyndromic hearing loss during a period in late childhood (approximately 10 years of age). Although children with these etiologies of hearing loss eventually catch up with peers with nonsyndromic hearing loss during adolescence, reduced speech perception in quiet during early childhood may have consequential implications for language, academic, and social development.

There are individual variables that were removed from the final model during the model optimization procedure, including year of first device implantation (to account for era effect), cochlear implant experience, race/ethnicity, biological sex, cochlear implant revision history, number of other complex diagnoses, and speech/language delay. The removal of these factors indicates that they do not contribute additional explanatory power in the final model. These factors were previously shown to explain pediatric cochlear implant users’ speech perception development in other studies.^57^ Their lack of significance in this study may reflect shared variance with other concurrently modeled individual, clinical, and environmental factors.

This study has some limitations. The current longitudinal clinical dataset is limited to speech perception testing in quiet. Although understanding speech in noise is an important aspect of real-world communication, it remains challenging to include in routine clinical assessment for pediatric cochlear implant users due to a lack of standardized signal-to-noise ratio to administer speech-in-noise tests. The current clinical dataset spans over 3 decades and was developed and managed by 2 co-authors, who ensured consistency in clinical standards for speech testing and data entry quality. Over time, multiple cochlear implant audiologists in training contributed to the testing records. As a result, minor variations and human errors may exist in the dataset; however, we anticipate no substantial impact on the data quality. Our discussion on the effect of SES was based solely on patients’ insurance type, which is a proxy and cannot fully represent the composite measure of SES. Future studies may consider adding other SES measures (eg, family zip code, income level, educational level) to verify the impact of SES on speech perception development. Additionally, nonhearing-related medical diagnoses cannot be temporally aligned to understand the effect of time-sensitive factors, such as the onset of comorbidities. Future work incorporating time-aligned longitudinal measures, speech perception in noise, family and educational support, and broader cognitive and language outcomes may further elucidate protective and risk factors shaping long-term cochlear implant outcomes.

## Conclusions

In this retrospective longitudinal cohort study, we identified multiple factors associated with speech perception in quiet among pediatric cochlear implant users, including daily device use, device configuration, SES, primary language, age at first device activation, vestibular involvement, and etiology of hearing loss. Two factors were consistently associated with slower developmental rate of speech-in-quiet perception in the first decade of life: (1) auditory neuropathy or syndromic hearing loss, and (2) low SES. Outcomes may be improved in these populations by improving access to pediatric cochlear implant specialists.^58^, ^59^, ^60^ Daily device use emerged as a key modifiable factor to promote speech-in-quiet perception development, particularly for later-implanted children, to mitigate developmental delays. Promoting consistent, full-time device use should continue to be an important target for intervention. Patients, caregivers, providers, and educators may benefit from additional counseling, education, and resources to encourage consistent device use,^61^^,^^62^ especially those who care for children with significant audiologic or medical factors associated with elevated risks for poor speech-in-quiet outcomes. Together, these findings provide clinically relevant guidance for multidisciplinary teams, including pediatricians, audiologists, speech-language pathologists, pediatric otolaryngologists, social workers, and deaf and hard-of-hearing educators, who support children with cochlear implants. Coordinated efforts across providers to promote consistent device use and equitable access to health care resources may help to improve long-term speech perception outcomes in pediatric cochlear implant users.

## CRediT authorship contribution statement

**Chieh Kao:** Writing – review & editing, Writing – original draft, Visualization, Validation, Software, Resources, Project administration, Methodology, Investigation, Formal analysis, Data curation, Conceptualization. **Kathryn B. Wiseman:** Writing – review & editing, Investigation, Formal analysis, Data curation. **Sophie E. Ambrose:** Writing – review & editing, Data curation. **Jacquelyn L. Baudhuin:** Writing – review & editing, Methodology, Data curation. **Adam K. Bosen:** Writing – review & editing, Investigation. **Amberlee Haggerty:** Writing – review & editing, Data curation. **Melissa R. Henry:** Writing – review & editing, Data curation. **Kristen L. Janky:** Writing – review & editing, Resources, Data curation. **Elizabeth A. Kelly:** Writing – review & editing. **Jessie N. Patterson:** Writing – review & editing, Data curation. **Jeffrey L. Simmons:** Writing – review & editing, Resources, Methodology, Investigation, Data curation, Conceptualization. **Victoria Sweeney:** Writing – review & editing. **Z. Ellen Peng:** Writing – review & editing, Writing – original draft, Visualization, Validation, Supervision, Software, Resources, Project administration, Methodology, Investigation, Formal analysis, Data curation, Conceptualization.

## Declaration of Competing Interest

The authors declare no conflicts of interest.
