# Supplementary material for: Speech Perception Development From Childhood to Adulthood Following Pediatric Cochlear Implantation: A 30-year Longitudinal Study
Source: J Pediatr Clin Pract. 2026 Apr 30;21:200213. doi: 10.1016/j.jpedcp.2026.200213 (PMC13234484; doi:10.1016/j.jpedcp.2026.200213)
Supplement: Supplementary Material 1 [file mmc1.docx]

**Supplementary Materials**

**Number of Visits Post Participants’ First CI Activation.** This figure shows that the Cochlear Implant (CI) Clinic at Boys Town National Research Hospital followed patients for 10 to 20 years after their first CI activation.


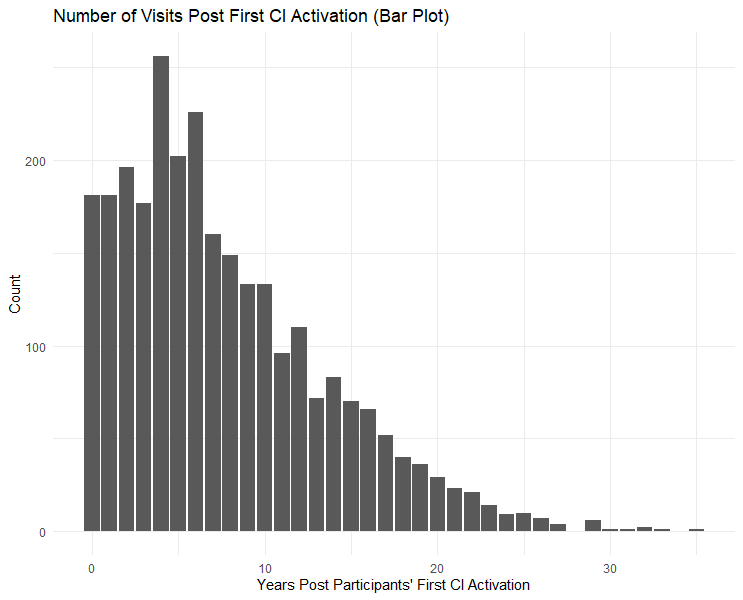


**Distribution of Follow-up Durations.** This figure shows that there are few participants who were lost to follow up (follow-up duration less than 1 year).


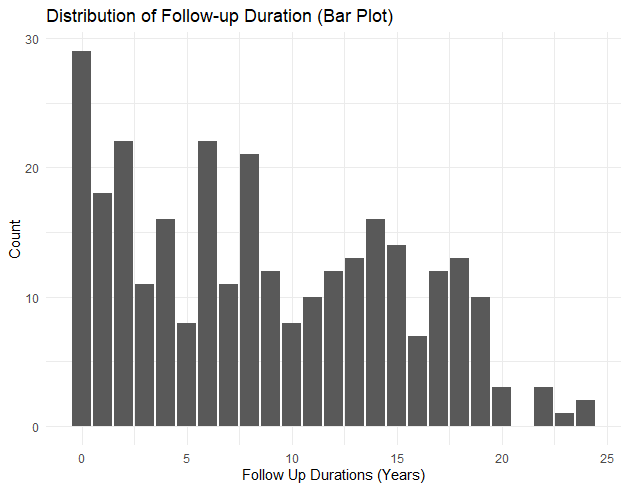


**There is no statistically detectable evidence of systematic differences based on variables of interests between participants who remained for long-term CI follow-up visits and participants who were lost to follow up within 1 year.**

We ran a multivariable logistic regression model to examine if any of the variable of interests in our primary analysis (audiologic, medical, device-related, and demographic) predicts participants who eventually were lost to follow up (follow up duration shorter than one year, N = 24/294). Because some variables produced non-estimable confidence intervals (very few cell counts in certain variables among the lost-to-follow-up group), we ran a Firth penalized logistic regression. None of the variables of interest were significantly associated with the patients who were eventually lost to follow-up, suggesting no statistically detectable evidence of systematic differences between early dropouts and those with longer follow-up. The results are reported as odds ratios with 95% confidence intervals in the following table.

| **Variables of Interest** | **OR** | **95% CI** | ***p* value** |
| --- | --- | --- | --- |
| Intercept | 0.69 | 0.001-359.8 | 1 |
| First CI Age of Activation (18-36 months) | 5.6 | 0.4-309 | 1 |
| First CI Age of Activation (after 36 months) | 4.07 | 0.44-287.15 | 1 |
| Etiology (Cochlear Nerve Deficiency) | 52.97 | 0.58-43212.93 | 1 |
| Etiology (Cochleovestibular malformations) | 0.3 | 0.001-12.7 | 1 |
| Etiology (Genetic Non-syndromic) | 0.29 | 0.0005-14.87 | 1 |
| Etiology (Genetic Syndromic) | 0.72 | 0.003-23.39 | 1 |
| Etiology (Multiple Etiologies) | 8.47 | 0.027-4023.36 | 1 |
| Etiology (Postnatal Infection) | 1.99 | 0.003-326.52 | 1 |
| Etiology (Prenatal Infection) | 0.62 | 0.004-18.72 | 1 |
| Etiology (Unknown) | 0.59 | 0.07-7.4 | 0.53 |
| Language (English) | 2.65 | 0.19-425.38 | 1 |
| Language (Spanish) | 5.1 | 0.17-954.32 | 1 |
| Daily Device Use | 0.57 | 0.26-1.1 | 0.1 |
| Device Configuration (bimodal) | 0.77 | 0.04-8.09 | 0.76 |
| Device Configuration (Single-Sided Deafness) | 0.28 | 0.002-6.29 | 1 |
| Device Configuration (Unilateral CI) | 0.98 | 0.004-15.17 | 0.84 |
| Diagnosis of Speech Delay | 1.08 | 0.005-20.57 | 0.6 |
| Vestibular Involvement | 0.58 | 0.03-3.01 | 1 |
| Complex Diagnoses | 1.02 | 0.11-6 | 0.83 |
| Gender | 1.23 | 0.17-10.59 | 1 |
| Race/Ethnicity (Caucasian) | 0.2 | 0.001-37.11 | 1 |
| Race/Ethnicity (Hispanic) | 0.29 | 0.0001-912.36 | 1 |
| Race/Ethnicity (Unknown) | 0.65 | 0.0008-695.57 | 1 |
| Insurance Type (Private) | 1.15 | 0.15-9.66 | 0.97 |
| Revision History | 0.63 | 0.01-5.57 | 1 |

*OR = odds ratio; CI = confidence interval
