# Supplementary material for: Speech Perception Development From Childhood to Adulthood Following Pediatric Cochlear Implantation: A 30-year Longitudinal Study
Source: J Pediatr Clin Pract. 2026 Apr 30;21:200213. doi: 10.1016/j.jpedcp.2026.200213 (PMC13234484; doi:10.1016/j.jpedcp.2026.200213)
Supplement: Supplementary Material 2 [file mmc2.docx]

Appendix: Dictionary of each variable included in the initial model

| **Predictor** | **Variable Name/Type/Unit** | **Levels or Range** | **Variable Description** |
| --- | --- | --- | --- |
| Chronological age | AgeAtTest_yr  Continuous  Year | 0.9 – 41.9 years | Age at the time of testing |
| Hearing age | PostCIStimAtTest  Continuous  Month | 0.1 – 34.6 years | Years after first CI activation |
| First CI age of activation | First_CI_3groups  Categorical | - Early = before 18 months - Middle = between 18 – 36 month - Late = after 36mo | Age at the first CI activation |
| First CI implanted year | FIRST_CI_YEAR  Continuous  Year | - 1989 – 2024 | Year when the participant received their first CI |
| Device configuration | CI_configuration  Categorical | - bilateral CIs - unilateral CI - bimodal (one CI and one hearing aid) - single-sided deafness with one CI | Daily listening device(s) |
| Daily device use | datalog_high  Continuous  Hours | 0.1 – 23.3 hours | Max datalogging hours from the most recent record (may be from a hearing aid in bimodal users) |
| Etiology of hearing loss | ETIOLOGY_TYPE  Categorical | - Auditory neuropathy - Congenital cytomegalovirus - Cochlear nerve deficiency - Cochleovestibular malformation - Non-syndromic genetic origin - Syndromic genetic origin - Postnatal infection - Multiple etiologies - Unknown | Etiologies of hearing loss identified through medical records review |
| Device revision history | RevisionHistory  Categorical | - 1 = Yes - 0 = No | History of explanation and re-implantation |
| Primary language | LANGUAGE_2  Categorical | - American Sign Language - English - Spanish | Primary language reported by the patients or their family in the medical records |
| Insurance type | INSURANCE  Categorical | - Government-based - Private | Insurance listed in the medical records |
| Race/Ethnicity | RACE_1  Categorical | - Asian - Black - Hispanic - White - Unknown | Race/ethnicity reported by the patients or their family in the medical records |
| Biological sex | GENDER  Categorical | - F = female - M = male | Biological sex documented in the medical records |
| Diagnosis of speech or language delay | Dx_SpeechDelay  Categorical | - 1 = Yes - 0 = No | History of diagnosis of receptive and/or expressive language delay |
| Diagnosis of vestibular involvement | VestibularCode  Categorical | - Nan = No vestibular evaluation records - 0 = Normal - 1 = Unilateral vestibular involvement - 2 = Bilateral vestibular involvement | History of vestibular evaluation and diagnosis of any vestibular involvement |
| Number of other complex diagnoses | Dx_Severity_4groups  Categorical | - 1 = None - 2= one other diagnosis - 3 = Two other diagnoses - 4 = More than three other diagnoses | History of other comorbid diagnoses documented in the medical records, including attention-deficit-hyperactivity disorder, autism spectrum disorder, behavioral disorders, motor disorders, receptive and/or expressive speech and language delay, vestibular or balance disorders, vision related disorders, and other developmental delay disorders. |
| Peabody Picture Vocabulary Test | PPVT  Continuous | Scores range from 51 to 139 | 43 out of 294 patients have PPVT scores available. |
| Wechsler Abbreviated Scale of Intelligence | WASI  Continuous | Scores range from 67 to 140 | 55 out of 294 patients have WASI scores available. |
